# Nodulocystic basal cell carcinoma on the limb masquerading as vascular malformation on imaging

**DOI:** 10.1093/jscr/rjac618

**Published:** 2023-01-10

**Authors:** Paul Brian Ng Hung Shin, Bibiana Tie, Vijay Kanagarajah, Ailin Tan

**Affiliations:** Department of General and Transplant Surgery, Princess Alexandra Hospital, Brisbane, QLD, Australia; Department of General and Transplant Surgery, Princess Alexandra Hospital, Brisbane, QLD, Australia; Department of General and Transplant Surgery, Princess Alexandra Hospital, Brisbane, QLD, Australia; Department of General and Transplant Surgery, Princess Alexandra Hospital, Brisbane, QLD, Australia

**Keywords:** basal cell carcinoma, nodulocystic, vascular malformation

## Abstract

Nodulocystic basal cell carcinoma (BCC) is a cystic variant of BCC, which can easily be misdiagnosed. We report a case of a 52-year-old man with a nodulocystic BCC that appeared at the site of a previously excised BCC 9 years prior. It examined as a benign cyst with a radiological signature of a vascular malformation. It was histologically confirmed on fine needle aspirate (FNA) and excisional biopsy to be a nodulocystic BCC. BCC is one of the most common paraneoplastic neoplasms affecting photo-exposed areas and displaying many variants. Nodulocystic BCC is a rarer variant that may have more than one dermoscopic face and can appear macroscopically benign. Given its malignant potential, it is imperative that it is accurately diagnosed. We highlighted that nodular cystic BCC can easily be misdiagnosed. Careful history and FNA are key differentiators to establish the correct diagnosis.

## INTRODUCTION

Recurrence of basal cell carcinoma (BCC) after initial treatment is not uncommon. Having its origin from follicular germinative cells, its histologic appearance may vary considerably depending on the direction and degree of differentiation of the cells [1]. We describe a case in which BCC developed cystic change leading to confusion given its clinical and radiological features.

## CASE PRESENTATION

**Figure 1:**
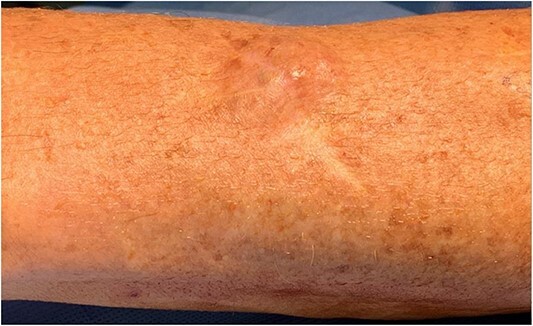
Clinical photograph of lump with the left side of image pointing distally towards thumb.

A 52-year-old man was referred by his general practitioner with a mass arising from his dorsal distal left forearm overlying the radius. On examination, he had a 4 × 3 cm, mobile, cystic mass (Fig. 1). Pertinent history includes two consecutive excisions of a nodular BCC 9 years ago at the same site, with histology showing nodular BCC.

**Figure 2:**
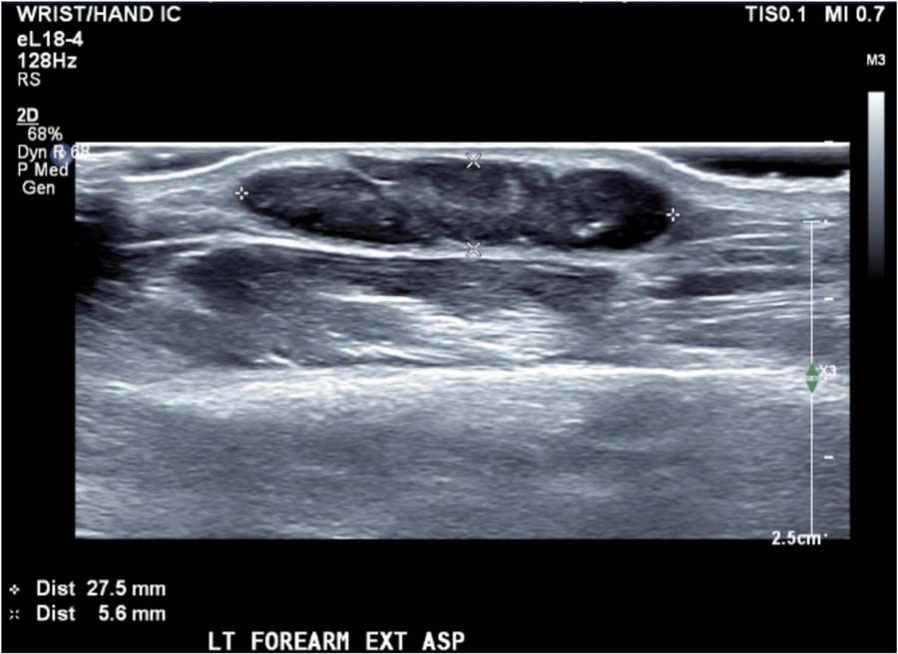
Ultrasound of left forearm with region of interest.

Ultrasound of the forearm revealed a 2.8 × 2.3 × 6 mm hypoechoic fusiform mass within the subcutaneous adipose tissue (Fig. 2). It was well marginated with relatively minor internal vascularity and with no extension into the deep soft tissue planes.

**Figure 3:**
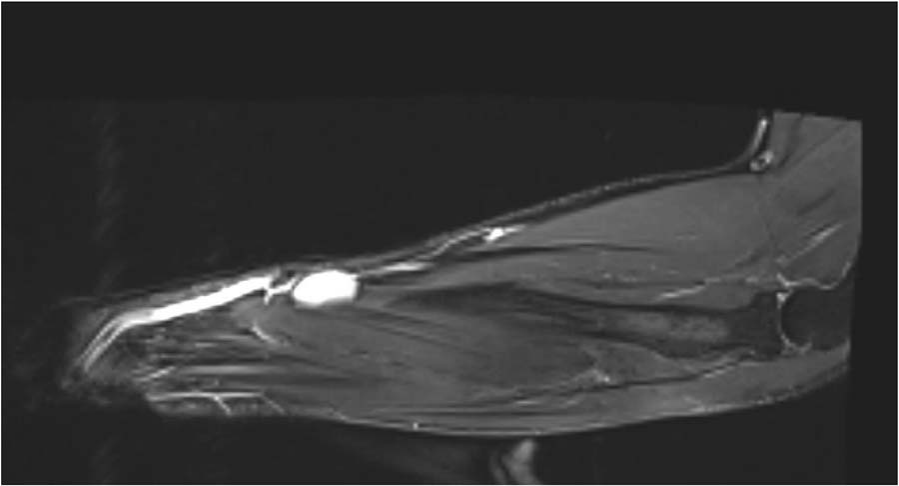
MR T1 coronal view of the left forearm showing concerning lump.

A subsequent MRI, showed a hyper intense 23 × 5 × 15 mm subcutaneous mass, with prominent adjacent feeding vessels, overlying the muscle bellies of abductor pollicis longus and extensor pollicis brevis (Fig. 3). Radiologically, it was thought to mimic the previous ultrasound and a differential of a vascular malformation/haemangioma was favoured.

Given the diagnostic dilemma, a fine needle aspirate biopsy was performed and showed the specimen to contain irregular groups of hyperchromatoid basaloid cells with peripheral palisading surrounding eosinophilic stroma in a background of myxoid matrix. Morphologically, the features were consistent with a recurrence of BCC.

**Figure 4:**
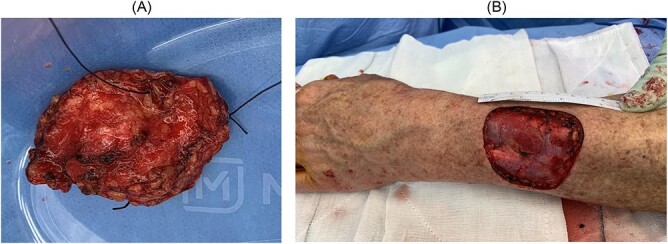
(**A**) Excision of lesion with cut undersurface in view with stitch at 12 o’clock; (**B**) surgical bed following excisional biopsy showing previous location of cut tumour.

The patient then proceeded to have a full thickness excision of the cystic lesion with margins down to the extensor muscles (Fig. 4A and B).

**Figure 5:**
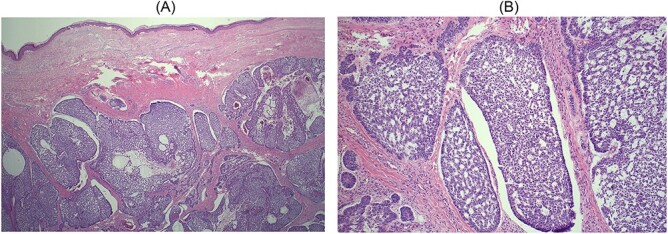
(**A**) Haematoxylin and eosin × 2. Low magnification view showing nodular nests of hyperchromatic basaloid cells with myxoid matrix, retraction artefact and peripheral nuclear palisading characteristic of BCC. There is also central cystic change because of degeneration of neoplastic cells; (**B**) haematoxylin and eosin × 10. Higher magnification of an area of cystic degeneration. Some of the cystic spaces are filled with red blood cells, mimicking vascular spaces.

Sectioning showed a 30 × 30 × 8 mm cream multiloculated mucoid nodule. On microscopy, the sections show Nodulocystic and focally infiltrative BCC that invades the subcutis with clear excision margin. No peripheral or lymphovascular invasion was seen (Fig. 5A and B).

The patient made a full recovery and was discharged from our clinic with recommendation of regular full skin check with his general practitioner.

## DISCUSSION

Clinical and morphological subtypes of BCC vary with the classic type being nodular. Tumoral necrosis in nodular BCC may lead to cavity formation and result in its cystic form [2].

Nodulocystic variant is not innocuous and has been described to have metastatic potential [3]. Henceforth, it is imperative to make a correct diagnosis.

The initial evaluation of BCC includes clinical examination, biopsy and imaging for staging. Imaging characteristic in this case was a red herring. Literature review in ultrasound and MRI characteristics of nodulocystic BCC yielded no directly relevant article. We presumed that this is because of the retrospective nature of cases with nodulocystic BCC.

The key differentiator in this case was the classical cytomorphological feature of BCC on fine needle aspiration cytology (FNAC). Cytological diagnosis with FNAC is reliable with a meta-analysis showing a pooled sensitivity of 97% (95% confidence interval (CI) 94–99) and pooled specificity of 86% (95% CI 80–91) [4].

## CONCLUSION

BCC has many histologic variants with a rare variant of nodular BCC being its cystic form. The importance of making a correct diagnosis lies in the necessity to differentiate this tumour from a benign cyst given its malignant potential. The knowledge of cytological features of the cyst is helpful to prevent any misdiagnosis.

## CONFLICT OF INTEREST STATEMENT

None declared.

## FUNDING

This research did not receive any specific grant from funding agencies in the public, commercial or not-for-profit sectors.

## Data Availability

All data has been included in this case report. Consent form can be requested as needed.
